# The dynamic influence of the DRB1*1101 allele on the resistance of sheep to experimental *Teladorsagia circumcincta *infection

**DOI:** 10.1186/1297-9716-42-46

**Published:** 2011-03-08

**Authors:** Musa Hassan, Barbara Good, James P Hanrahan, Deirdre Campion, Gearoid Sayers, Grace Mulcahy, Torres Sweeney

**Affiliations:** 1 School of Agriculture, Food Science and Veterinary Medicine, University College Dublin, Ireland; 2 Teagasc, Animal Production Research Centre, Athenry, Co. Galway, Ireland; 3 Department of Biology, Massachusetts Institute of Technology, Cambridge, Massachusetts, USA

## Abstract

Suffolk sheep carrying the DRB1*1101 (previously referred to as-DRB1*0203 or G2) allele have been reported to show increased resistance to natural *Teladorsagia circumcincta *infection compared to non-carriers. The objective of this study was to compare the biochemical and physiological responses of DRB1*1101 carrier and non-carrier twin lambs to an experimental infection with 3 × 10^4 ^L3 *Teladorsagia circumcincta*. The variables studied included worm burden, faecal egg count, abomasal mast cells, IgA, IgE, IgG1 plus IgG2 and haematological parameters at 0, 3, 7, 21 and 35 days post infection (dpi), and duodenal smooth muscle contractility at 0 and 35 dpi. DRB1*1101 carrier lambs had significantly lower worm burden, higher mast cell and plasma platelet counts than the DRB1*1101 non-carriers (*P *< 0.05). Before infection, the non-carrier lambs exhibited significantly higher mucosal levels of all antibody isotypes measured compared to the carriers; these levels remained relatively stable over the course of infection in the non-carriers while there was a slow build up of these antibodies in the carriers up to day 21 post infection (pi). The DRB1*1101 non-carrier lambs had a significantly higher plasma lymphocyte count, and produced greater duodenal contractile force relative to the carrier lambs (*P *< 0.05). There was no significant difference between genotypes in the level of plasma eosinophils, monocytes, neutrophils or FEC. This evidence suggests that resistance conferred by DRB1*1101 is acquired rather than innate, depends on worm expulsion rather than fecundity and is dependent on mucosal mast cell proliferation, platelet activation, and IgA and IgE antibody responses.

## Introduction

The abomasal worm *Teladorsagia circumcincta *(*T. circumcincta*) is amongst the most important parasites affecting livestock production [1,2] and predominates in cool temperate regions [3,4]. While control of these parasites has been achieved by the use of anthelmintic drugs, public demand for meat products free of chemical residues and the emergence of parasites resistant to anthelmintics [5-7], has made reliance on these drugs problematical. Genetic selection for parasite resistance in domestic sheep can help to reduce reliance on chemoprophylaxis. To this end, several genetic markers of resistance have been identified, including an allele (DRB1*1101) (also known as G2 and DRB1*0203) of the major histocompatibility complex DRB1 (MHC-DRB1) locus [8-10]. However, the biological mechanisms by which these markers exert their influence are not known.

Ovine resistance to *T. circumcincta *develops over time [11,12] and is dependent on the activation and development of a Th2 immune response, characterised by the production of anti-inflammatory cytokines such as interleukin (IL)-4, IL-5, IL-9, IL-13 [13-15], recruitment of mast cells and eosinophils in the mucosa [16-18], production of IgA [19-22] and IgE [23]. It is known that proper antigen presentation is essential to the development of a successful immune response [24]. Therefore, owing to the importance of the MHC molecules in antigen recognition and presentation [25-27], and the fact that an allele of the MHC-DRB1 locus has been associated with increased nematode resistance in sheep [10,28,29], it is plausible that MHC genes play a key role in determining the outcome of the immune response and nematode infection in general. Particularly, polymorphisms within the DRB1 locus, that encode the β1 domain of the MHC-II molecule which constitutes the peptide binding region [30], are likely to play a pivotal role in disease outcome.

A significant role for humoral immunity in ovine resistance to gastrointestinal nematodes has previously been reported [19,21,31]. Higher levels of IgG1 have been observed in the lymph of sheep infected with *T. columbriformis *[32], suggestive of a role for this antibody in nematode resistance. Similarly, elevated levels of IgA have been observed in lymph of sheep undergoing *T. circumcincta *infection [20,31,33], while IgA-assisted suppression of worm fecundity and arrested L4 development has been reported to mediate resistance in *T. circumcincta *infected sheep [19,21]. Additionally, a correlation between infection pathology and total serum IgE levels [34-38] and a polymorphism in the IgE gene [39] have been observed in sheep undergoing nematode infection. However, it is not clear how polymorphisms at the MHC locus affect the humoral immune response to gastrointestinal nematodes.

In the present study, a group DRB1*1101 carrier and non-carrier twin lambs, was used to investigate the relationship between this locus and the physiological and immunological processes driving resistance to *T. circumcincta *such as, smooth muscle contractility, abomasal mast cell hyperplasia, and production of IgA and IgE.

## Materials and methods

### Animals

Six unrelated sires heterozygous for the DRB1*1101 allele were mated with ewes in single sire groups to generate progeny carrying or lacking the DRB1*1101 allele. All lambs were genotyped at the DRB1*1101 locus, as previously described [28,40]. This allele was initially named G2 [10,29] and Ovar-DRB1*0203 [8]. However due to the international standardisation of the nomenclature of the MHC alleles, this allele is now known as the DRB1*1101 [41]. It was intended that lambs used in the study would be twin pairs where one sibling was a carrier of the DRB1*1101 allele and the other sibling a non-carrier. Within the constraints of birth date, birth type and lamb survival, 18 suitable twin pairs were identified. These were randomly assigned to 1 of 5 slaughter dates (days 0, 3, 7, 21, and 35 of the experiment) subject to the constraints that there were a maximum of four pairs per slaughter date. To maintain numerical balance in the design, we utilised a twin pair where both siblings were carriers on day 3 and another pair where both siblings were non carriers on day 21. Even though the ewes were not genotyped, since all the sires were heterozygotes at the DRB1 locus we could identify the source of the DRB1*1101 allele where the second allele did not match the sire second allele, however, where both the alleles in the progeny matched the sire's alleles (1 lamb) we could not tell the origin of the DRB1*1101 allele. The lambs were born indoors at the Teagasc Animal Production Research Centre, and were put to pasture until they were between 4 and 5 weeks of age when they were moved back indoors with their dams. All lambs were weaned 1 week later and faecal sampled to determine faecal egg count 3 weeks after weaning (80% of the lambs had a FEC < 150 and the maximum FEC observed was 250) and treated with Oramec (Merial Animal Health Ltd, CM19 5TG, Harlow, UK) as per the manufacturer's instructions. The lambs were moved to Lyons Research Farm, University College Dublin, at about 10 weeks of age, housed in a nematode free environment and given two weeks to acclimatise prior to the start of the experiment. The lambs were free of nematode infection at the start of the experiment, based on FEC measurements on three consecutive days. All procedures described in this experiment were conducted under experimental licence from the Irish Department of Health in accordance with the Cruelty to Animals Act 1876 and the European Communities (Amendments of the Cruelty to Animals Act, 1976) Regulations, 1994. The lambs received granulated feed and water *ad libitum*. At about 12 weeks of age, eight lambs were slaughtered (day 0; controls) and the remaining lambs each received a single oral dose of 3 × 10^4 ^*T. circumcincta *L3. The subsequent slaughter dates were chosen based on the developmental stages of the parasite in the sheep abomasum; day 3 (L3 stage), day 7 (L4 stage) and days 21 and 35 (egg laying stage).

### Worm count

Following necropsy on 0, 3, 7, 21, and 35 days post infection (dpi), the abomasum was recovered and the contents collected, washed through two sieves (75 μm and 38 μm) and preserved in 10% neutral buffered formaldehyde for worm recovery. Each abomasum was then dissected along its greater curvature and one complete fold was removed just distal to the midline of the abomasum, and rinsed briefly in warm physiological saline. The recovery of worms from the abomasal mucosa was performed using a saline digest technique [42]. Both larval and adult worms from the abomasum contents and digests were counted. Worm burden was calculated by extrapolation from a 2% aliquot count.

### Faecal egg count

Faecal samples were collected, on day 23, 28, 31, and 35, from the rectum of the lambs designated for slaughter on day 35 post infection (pi). The samples were then used for faecal egg count determination using the modified McMaster method [43].

### Blood sampling

Blood samples were collected into vacutainers, with EDTA, immediately prior to slaughter by jugular venepuncture. Plasma was collected by centrifuging at 2 000 *g *for 5 min at 4°C and stored at -80°C until use.

### Mucosal collection and antibody recovery

At necropsy, the surface layer together with mucus epithelial layer were removed by scraping with a blunt blade, placed in cryovials and snap frozen in liquid nitrogen. The samples were then stored at -80°C until use. Mucosal samples were prepared for antibody recovery as described by Sinski et al. [44]. Briefly, thawed samples were homogenised using a Retsch^® ^tissuelyser (Qiagen, Crawley, UK) in 3 volumes of phosphate buffered saline (PBS) containing 5 μg/mL of protease inhibitor cocktail (Sigma, Wicklow, Ireland). The homogenate was centrifuged at 12 000 *g *for 30 min and the supernatant stored at -20°C until use. Prior to storage, an aliquot of the supernatant was taken and for the determination of protein concentration using the BCA protein assay reagent kit (Pierce, Rockford, IL, 61105, USA).

### Larval antigen preparation

Third stage larvae (L3) were prepared as described previously [44]. *T. circumcinta *L3 (1 × 10^6^) obtained from faecal cultures were cleaned extensively by centrifugation, filtered through saturated sucrose solution and washed in PBS. Larvae were homogenised using 180 μm diameter glass beads (Sigma) for 60 min. The homogenate was repeatedly freeze-thawed 10 times and stored at 4°C overnight after addition of 5 mg/mL of protease inhibitor cocktail (Sigma). The homogenate was centrifuged at 14 000 *g *for 60 min and the supernatant passed through a 0.22 mm diameter filter membrane. Protein concentration was determined using the BCA protein assay kit (Pierce), adjusted to 250 μg then stored at -20°C until use.

### Enzyme-linked immunosorbent assay

The procedure used has previously been described [23,34]. Polystyrene ELISA plates were coated with 100 μL of L3 antigen (5 μg/mL) in carbonate buffer at pH 9.6 and stored overnight at 4°C. Plates were washed 4 times in PBS containing 1% Tween (PBS-T) and a 100 μL aliquot of either mucosal sample (adjusted to 500 μg/mL in PBS-T/3% BSA) or serum (diluted 1:1000, 1:100, 1:50 for IgG1, IgG2 and IgA, and IgE respectively) was added to each well and incubated at 37°C for 30 min. In the case of IgE, the mucosal and plasma sample were pre-heated to 56°C [36]. Plates were then washed 4 times in PBS-T and 100 μL of monoclonal mouse anti-bovine IgG1 plus IgG2 (AbD Serotec, Oxford, UK) diluted 1/1000, monoclonal mouse anti-ovine IgA (AbD Serotec), diluted 1/50 and monoclonal mouse anti-sheep IgE (received from Prof. Frans Kooyman, Utrecht University, The Netherlands), diluted 1/1000, was added for each assay and incubated at 37°C for 30 min. Plates were then washed in PBS-T and 100 μL of a secondary goat antimouse-horse radish peroxidise (HRP) conjugate (Dako diagnostics, Dublin, Ireland) was added and incubated at 37°C for 30 min. After four washes in PBS-T, 100 μL of chromogen tetramethylbenzidine (TMB) (Dako diagnostics) was added and incubated at room temperature for 15 min and the reaction stopped in 10% 1 M HCL. The optical density of each plate was read at 450 nm in an Asys™ UVM-240 microplate reader (VWR International, Dublin, Ireland). All plates contained a blank (PBS instead of plasma) plus a negative and a positive control (single serum sample yielding low and high level anti-nematode specific antibodies, respectively).

### Mast cell count

After the recovery of the contents from the abomasum, a small piece (~ 2 cm) was cut from the abomasal midline fold and fixed in 10% neutral buffered formalin. Samples were then processed in a Sakura Tissue-Tek^® ^VIP processor (Clinical distributor, Dublin, Ireland). After processing, the samples were sectioned using a Leitz^® ^1512 Microtome (GMI Inc, Ramsey, Minnesota, 55303, USA), mounted on microscopic slides. The mast cells were then stained using the uranyl nitrate metachromatic method [45]. Sections were examined using QImaging colour camera (Nikon, Japan) under a ×40 objective and the images visualised using Image-Pro program. Under this magnification, a field area of 0.023 mm^2 ^was used to count mast cells. A total of 10 such fields were counted per slide and a mean figure obtained. The counts were expressed as total number per 1 mm^2^.

### Haematology measurements

Blood samples were subjected to standard clinical procedures to determine the number of monocytes, platelets, lymphocytes, eosinophils, and neutrophils. Blood cell count was done in an ADVIA^® ^2120 haematology system (Bayer Healthcare, Leverkusen, Germany) as per manufacturer's recommendations.

### Measurement of smooth muscle contractile response

Two 1 cm sections of the duodenum, beginning approximately 1 cm from the sphincter and proceeding distally, were removed and placed in oxygenated (95% O_2 _and 5% CO_2_) Krebs solution. The lumen of each section was flushed gently with Krebs buffer. Longitudinal strips (full thickness, approximately 10 mm by 3 mm) were dissected and mounted vertically in a 15 mL water-jacketed baths pre-filled with oxygenated (95% O_2 _and 5% CO_2_) Krebs solution containing 118 mM NaCL, 11.1 mM D-Glucose, 24.9 mM NaHCO_3_, 1.2 mM MgSO_4_, 4.7 mM KCL, 1.2 mM KH_2_PO_4_, and 2.5 mM CaCL_2 _and maintained at 37°C. A resting pre-load of 0.5 g was applied to each muscle strip, and then allowed to equilibrate for 20 min. Mechanical activity was recorded using isometric force transducer (World Precision Instruments, Stevenage, Herts, UK). Tension was monitored continuously and recorded using a MacLab™ data acquisition system (AD Instruments Ltd, Hastings, UK). The optimum tension was chosen following repeated measurements whereby the tissues were stretched by the application of increasing force, equivalent to 0.25 mg, and the contraction was assessed after stimulation with the synthetic muscarinic agonist carbachol (Sigma-Aldrich, Dublin, Ireland) followed by wash-out, 15 min equilibration, and a further application of an increment of tension and stimulation.

For the full experiment, two strips of tissue from each animal were exposed to a cumulative concentration of 1 nM to 1 mM carbachol by addition of aliquots to the baths. After the final addition of the drug, the tissues were rinsed, blotted with tissue paper, and weighed. The results were expressed as units force per unit tissue weight (mN/mg).

### Statistical analyses

Prior to analysis, counts (X) were transformed to logarithms (log_e_(X + 25) for FEC and log_e_(X + 1) for worm counts) to stabilise the variance. The haematological data and the OD values from antibody measurements were subjected to square root transformation prior to analysis. All data were analysed using SAS procedures, by fitting a model that had effects for genotype, dpi (time) and their interaction for variables measured at time point. In the case of FEC (day 35 group only) the model had genotype and sample day as fixed effects and animal within group as random term to accommodate the repeated nature of the measurements.

For each animal, the mean duodenal tension was calculated from the duplicates and expressed as mean ± s.e. Statistical significance was calculated using student's *t*-test to compare the means of carrier and non-carrier lambs where a significant genotype by time interaction was detected.

## Results

### Worm count

**Figure 1:**
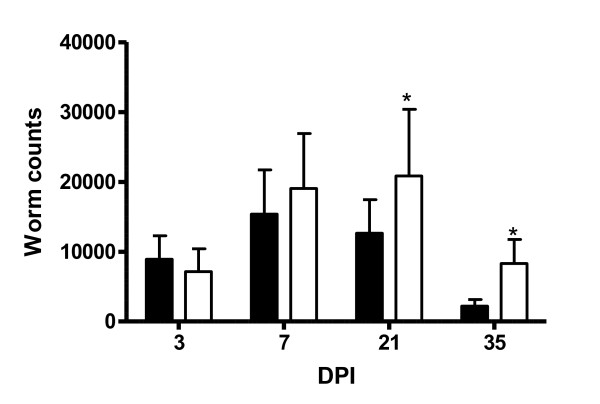
Worm burden (mean ± ci) in the abomasum of DRB1*1101 carriers (black bars) and non-carriers (white bars) lambs following infection with 3 × 10^**4 **^***T.circumcincta *L3**.

Mean worm counts from the DRB1*1101 carriers and non-carriers are presented in Figure 1. The DRB1*1101 carrier lambs had significantly lower worm count than the non-carrier lambs (*P *< 0.05). There was a significant (*P *< 0.001) time effect on the number of worms, and the genotype by time interaction was significant (*P *< 0.05). The peak number of worms recovered was on day 7 in the carriers and on day 21 in the non-carrier lambs

### Faecal egg count

**Figure 2:**
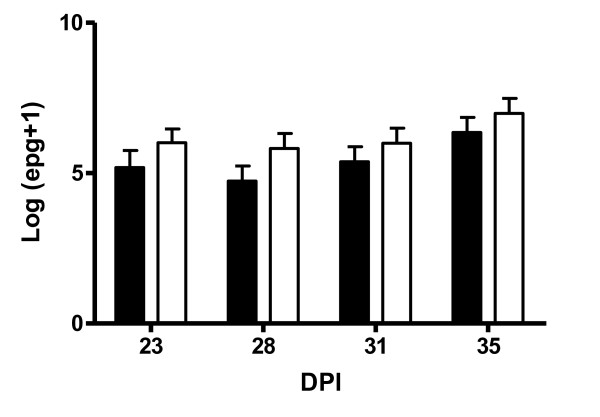
Shows the log transformed FEC from DRB1*1101 carrier (black bars) and non-carrier (white bars) lambs following infection with 3 × 10^**4 **^***T. circumcincta *L3**.

The average log-transformed FEC are presented in Figure 2. Time of measurement had a significant (*P *< 0.05) effect on FEC with the highest counts being observed on day 35. While the DRB1*1101 carrier lambs had numerically lower FEC than the non-carriers at each sampling time point, this difference was not significant and there was no significant genotype by time effect.

### Nematode specific mucosal antibody response

**Figure 3:**
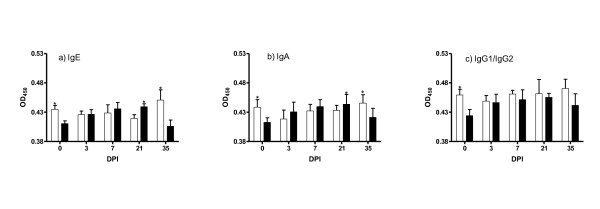
Mean OD values (mean ± s.e) of IgE, IgA and IgG1 plus IgG2 in the abomasal mucosa of DRB1*1101 carriers (black bars) and non-carriers (white bars) following infection with 3 × 10^**4 **^***T.circumcincta *L3**.

The mucosal antibody levels in the DRB1*1101 carrier and non-carrier lambs at each time point are presented in Figure 3. The genotype by time interaction was significant for IgA (*P *< 0.05), IgE (*P *< 0.05), and IgG1 plus IgG2 (*P *< 0.05). Before infection, the non-carriers had significantly (*P *< 0.05) higher levels of IgA, IgE and IgG1 plus IgG2 compared to the carriers. Following infection, there was a gradual build up in the levels of all antibody isotypes up to day 21, before a decrease on day 35 in the carrier lambs. In the non-carrier lambs, the levels of these antibodies remained relatively constant over the course of infection. Carrier lambs had significantly (*P *< 0.05) higher levels of IgA and IgE on day 21 than the non-carriers. The non-carriers had significantly (*P *< 0.05) higher levels of IgE and IgA on day 35 than the carriers. No significant differences were observed in IgG1 and IgG2 responses between the carriers and the non-carriers following infection.

### Nematode-specific plasma antibody response

**Figure 4:**
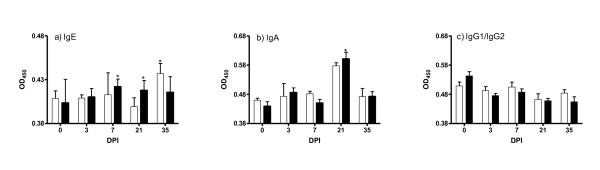
Mean OD values (mean ± s.e) of IgE, IgA and IgG1 plus IgG2 in the plasma of DRB1*1101 carriers (black bars) and non-carriers (white bars) following infection with 3 × 10^**4 **^***T.circumcincta *L3**.

The mean OD values for the antibody isotypes in the plasma at each time point are presented in Figure 4. There was significant genotype by day effect for IgA (*P *< 0.05) and IgE (*P *< 0.05). Significantly (*P *< 0.05) higher plasma level of IgE was observed on days 7 and 21 in the carriers compared to the non-carriers. The non-carriers had significantly (*P *< 0.05) higher levels of IgE on day 35. The carrier lambs had significantly (*P *< 0.05) higher plasma level of IgA on day 21. There were no significant differences in the values for IgG1 plus IgG2.

### Mast cell count

**Figure 5:**
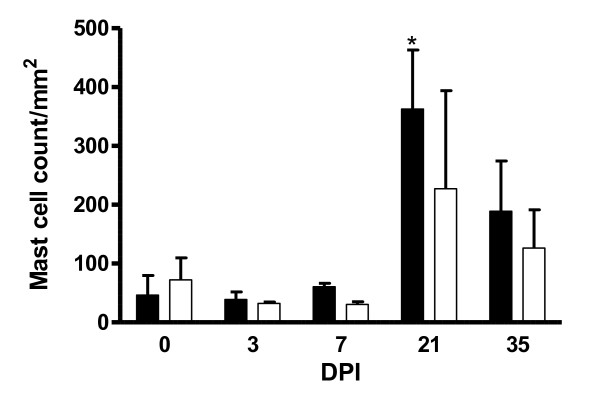
**Mean mast cell count per mm**^**2 **^(mean ± s.e) in the abomasum of DRB1*1101 carrier (black bars) and non-carrier (white bars) following infection with 3 × 10^**4 **^***T. circumcincta *L3**.

Mean mast cell counts for carriers and non-carriers are presented in Figure 5. There was a significant day effect (*P *< 0.05) on the number of mast cells, with a marked increase occurring on day 21 pi followed by a decrease on day 35 pi in both groups of lambs. The genotype by day interaction was also significant (*P *< 0.05). Significantly (*P *< 0.05) higher mast cell counts were obtained for carrier lambs from day 21 pi relative to the non-carrier lambs.

### Haematological measurements

**Figure 6:**
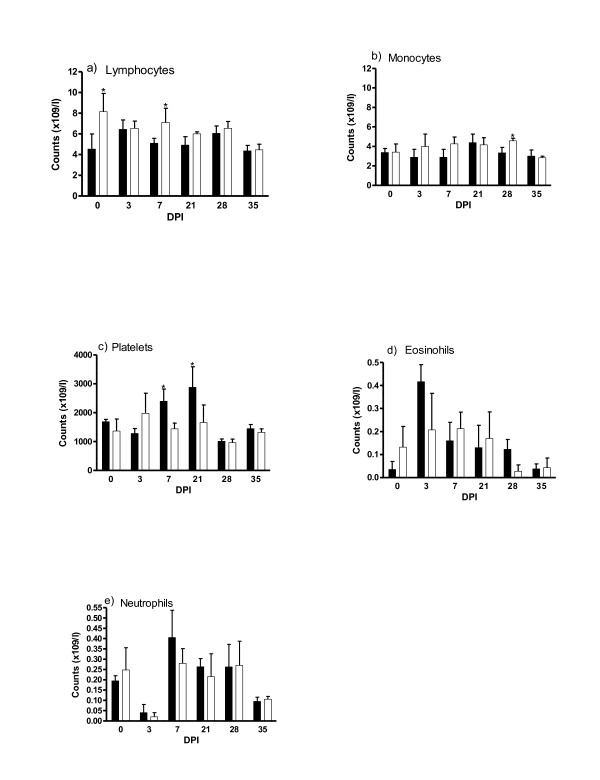
Haematological measurements in DRB1*1101 carrier (black bars) and non-carrier (white bars) lambs following infection with 3 × 10^**4 **^***T. circumcincta *L3**.

The mean circulating levels of lymphocytes, eosinophils, monocytes, neutrophils and platelets from the carrier and non-carrier lambs are presented in Figure 6. There was a significant (*P *< 0.05) day effect for all these cells counts. The genotype by day interaction was significant for lymphocyte (*P *< 0.05), monocytes (*P *< 0.05), and platelets (*P *< 0.05). Non-carrier lambs displayed significantly (*P *< 0.05) higher levels of plasma lymphocytes on days 0 and 7, and monocytes on day 28 pi than the carrier lambs. On the other hand, the carrier lambs had significantly (*P *< 0.05) higher levels of platelets on days 7 and 21 than the non-carriers. The number of eosinophils and neutrophils were not affected by the genotype.

### Muscle contractility

**Figure 7:**
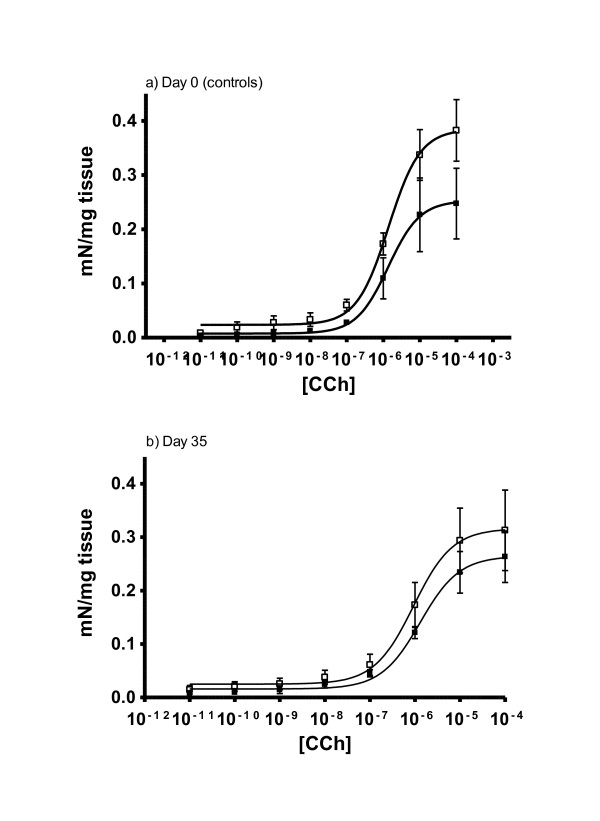
Response of duodenal smooth muscles from DRB1*1101 carriers (black boxes) and non-carriers (white boxes) infected with 3 × 10^**4 **^***T. circumcincta *L3 to increasing concentration of carbachol before (a) and at 35 dpi (b)**.

The dose-response relationship for carbacol-induced contraction in uninfected (day 0) and day 35-post infected carrier and non-carrier lambs are shown in Figure 7. In both groups, the response to carbachol was maximal at 10^-4 ^M. Before infection (day 0), the non-carrier lambs produced significantly (*P *< 0.05) greater contractile force than the carrier lambs.

**Figure 8:**
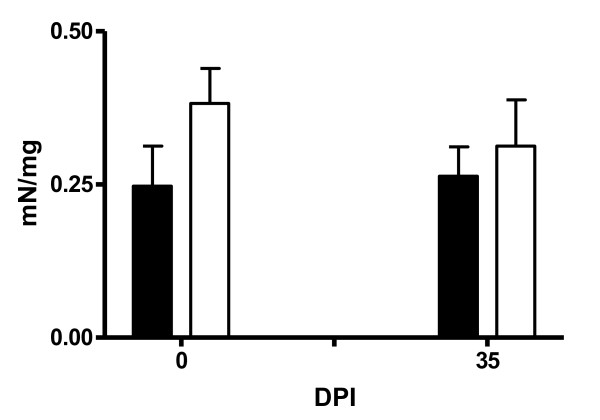
The response of the duodenal smooth muscles from DRB1*1101 carriers (black bars) and non-carriers (white bars) infected with 3 × 10^4 ^*T. circumcincta *L3 to carbachol at a concentration of 10^-4^.

The contractile response of duodenal smooth muscle to carbachol at 10^-4 ^M is presented in Figure 8. There was a genotype effect in the response generated at this dose with the non-carriers generating greater contractile force than the carrier lambs at 0 and 35 days pi (*P *< 0.05).

## Discussion

Possible confounding effects of genetic background and maternal influences were minimised in this study by using predominantly twin pair lambs consisting of an DRB1*1101 carrier and non-carrier. Genotype effect was detected for worm burden, mucosal IgE, IgA production, mucosal mast cell number, and plasma platelet count. The carrier lambs had significantly lower worm burden, higher levels of mucosal IgA, IgE and mast cell count. Interestingly, the highest mucosal IgE and IgA levels coincided with a reduction in worm count in both groups of lambs.

Humoral immunity is reported to play a significant role in resistance to nematodes [21,23,31,34,46] and the mucosal levels of IgE observed in this study are consistent with these observations. The gradual build up of plasma IgE levels in the DRB1*1101 carriers is similar to previous findings made in sensitised sheep undergoing *T. columbriformis *infection [47], indicative of a role for this antibody in the resistance as conferred by this allele. Noteworthy, the levels of IgE peaked on day 21, coinciding with a decrease in worm burden in these lambs. The fall to pre-infection levels of IgE on day 35 in the abomasal mucosa of DRB1*1101 carriers may be due to the need for these lambs to dampen the aggressive immune response as the worm burden in the abomasum declines and to redirect nutrients to other biological processes. The abomasal mucosa IgA levels varied through the course of infection in both the carrier and non-carrier lambs and are consistent with the proposed role for this antibody in nematode resistance. IgA has been associated with parasite loss, arrested larval development at the L4 stage [19-22,48] and reduced fecundity and worm length [21,49], possibly in conjunction with eosinophils [31].

The observations made on mast cells in the current study are consistent with findings in other independent studies and their associated role in increased resistance to nematode infection [16,49,50]. Mast cells have been reported to control worm burden [48,49], accounting for about one-third of the variation of worm numbers in deliberate *T. circumcincta *infections [49]. These observations perhaps explain the high mast cell number in the carrier lambs which coincided with reduced worm burden. The slow mast cell response observed in the current study can partly be explained by their involvement in protein loss and reduced growth of the host [51]. Similarly, the lack of an association between levels of eosinophils and increased resistance, in the current study, is supported by observations made previously [17,23,52]. However, other studies have shown a close association between eosinophils and the number of *H. contortus *larvae [52] but not *T. circumcincta *[23,31] in sheep, indicative of different mechanisms involved in *H. contortus *and *T. circumcincta *resistance. Consequently, the role of eosinophils in nematode resistance may depend on the parasite species and age of sheep undergoing infection. The high level of platelets in the DRB1*1101 carriers is interesting since platelets have been shown to be involved in parasitic infection [53]. They release various inflammatory mediators, are capable of phagocytosis and interact with other cells of the immune system [54,55]. It is noteworthy that the high platelet count coincided with the end of high worm burden in the abomasum of DRB1*1101 carrier lambs.

The significant influence of DRB1*1101 allele on the number of worms recovered from the abomasum in the current study are consistent with observations made following natural infection [51]. While a correlation between FEC and parasite numbers has been reported in other studies [49,56], the lack of correlation between FEC and worm numbers, in the present study, could be due to density dependent constraints on fecundity [49,57-59], which could emerge as more larvae develop into egg-laying adults. As the number of parasites within the host increase, there is a decline in the estimated average number of eggs produced per worm, which partly explains the stability of egg counts over time [58,60]. Since these lambs received a large single dose (3 × 10^4^) of infective larvae, density dependent constraint on fecundity is plausible. It is noteworthy, in this context that FEC increased while the worm count decreased from day 21 pi in the present study. However, the small number of lambs (4 per genotype) available in this study would have made it difficult to detect differences in FEC between the groups.

The high duodenal contractile force observed in the DRB1*1101 non-carriers relative to carriers contradicts observations made in murine models where increased gut smooth-muscle contractility is associated with resistance to gastrointestinal nematodes [61]. It is not known, however, if similar smooth muscle functions underlie parasite expulsion in sheep. Furthermore, contractile force was measured in the duodenum while the lambs were infected with an abomasal nematode and this may partly explain the current observation since the relationship between the contractile force generated in the duodenum and the abomasum is not known. Perhaps, the low contractility in the duodenum of carrier lambs is an adaptive process in these lambs aimed at increasing nutrient absorption in this parasite-free region [62], by increasing the time of contact between the digesta and epithelial layer. Consequently, further experiments need to be done to define the basis for the difference observed in the present study. It is also worth noting that while the mouse model has provided insight into some aspects of general immune response to GI nematodes, studies on mouse models cannot necessarily be applied to sheep, as evidenced by the very different role eosinophils play in nematode infection in the two models [31,63-65].

Overall, the association of the Ovar-DBR1*1101 allele with immunological parameters controlling *T. circumcincta *infection in the current study validates this allele as a marker of resistance. However, it does not answer the question of how variability at this locus controls resistance. Because DRB1 locus encodes the antigen recognition site of the MHC-II molecule, it would be expected that structural variations at this locus would confer variability in antigen recognition and processing [30], hence the basis of response variability in the carriers and non-carriers to *T. circumcincta *in the current study. Nevertheless, studies investigating antigen recognition amongst the ovine DRB1 alleles did not indicate any obvious association between specific parasite antigens and the Ovar-DRB1 alleles or vice versa [66]. However, the heterogeneity in antigen recognition by sheep antibodies [66] could have been due to modulation of immune repertoire by factors such as cross-tolerance, caused by polymorphisms in other genes which may mask simple immune repertoire and MHC alleles [67,68]. The strong linkage disequilibrium between the MHC genes makes it almost impossible to identify a single gene within this locus as the disease allele and it could be that the DRB1*1101 allele is linked to alleles at other loci which cause differential antigen recognition between the carriers and non-carriers. Consequently, antigen recognition studies based on DRB1 haplotypes rather than alleles would identify any differences in antigen recognition between the carriers and the non-carries, and by extension which haplotypes provide the best protection against *T. circumcincta*. On the other hand, resistance could be due interaction with a set of parasite proteins, rather than a single parasite epitope [66], therefore, an association between MHC haplotypes and a set of parasite epitopes would be an alternative way to understanding of the molecular networks involved in *T. circumcincta *resistance.

In conclusion, evidence from this study suggests that the mechanisms underlying nematode resistance conferred by the DRB1*1101 allele, is mediated by mast cell proliferation in the mucosa, and higher IgE, IgA and blood platelet levels. Additionally, the lower abomasal worm burden in the carriers, indicate that the low FEC associated with DRB1*1101 allele is due to an effect on worm establishment and expulsion rather than on fecundity.

## Competing interests

The authors declare that they have no competing interests.

## Authors' contributions

JP and TS conceived of the study. MH, JP, TS, BG designed the experiments. BG and MH collected samples. MH carried out the experiments and drafted the manuscript. GM helped design the immunological experiments. BG and JP analysed the parasitological data. GS helped with the DNA sequence analysis. DC performed the smooth muscle contractility experiments. All authors read and approved the final manuscript.
